# Generating a conformational landscape of ubiquitin chains at atomistic resolution by back-mapping based sampling

**DOI:** 10.3389/fchem.2022.1087963

**Published:** 2023-01-10

**Authors:** Simon Hunkler, Teresa Buhl, Oleksandra Kukharenko, Christine Peter

**Affiliations:** ^1^ Department of Chemistry, University of Konstanz, Konstanz, Germany; ^2^ Max Planck Institute for Polymer Research, Mainz, Germany

**Keywords:** molecular dynamics simulations, dimensionality reduction, back-mapping, coarse graining, clustering, ubiquitin, polyubiquitin

## Abstract

Ubiquitin chains are flexible multidomain proteins that have important biological functions in cellular signalling. Computational studies with all-atom molecular dynamics simulations of the conformational spaces of polyubiquitins can be challenging due to the system size and a multitude of long-lived meta-stable states. Coarse graining is an efficient approach to overcome this problem—at the cost of losing high-resolution details. Recently, we proposed the back-mapping based sampling (BMBS) approach that reintroduces atomistic information into a given coarse grained (CG) sampling based on a two-dimensional (2D) projection of the conformational landscape, produces an atomistic ensemble and allows to systematically compare the ensembles at the two levels of resolution. Here, we apply BMBS to K48-linked tri-ubiquitin, showing its applicability to larger systems than those it was originally introduced on and demonstrating that the algorithm scales very well with system size. In an extension of the original BMBS we test three different seeding strategies, i.e. different approaches from where in the CG landscape atomistic trajectories are initiated. Furthermore, we apply a recently introduced conformational clustering algorithm to the back-mapped atomistic ensemble. Thus, we obtain insight into the structural composition of the 2D landscape and illustrate that the dimensionality reduction algorithm separates different conformational characteristics very well into different regions of the map. This cluster analysis allows us to show how atomistic trajectories sample conformational states, move through the projection space and in sum converge to an atomistic conformational landscape that slightly differs from the original CG map, indicating a correction of flaws in the CG template.

## 1 Introduction

Nowadays molecular dynamics (MD) simulation is a well established tool to investigate proteins and protein complexes at atomistic resolution. However it can still be computationally very expensive to obtain convergent MD trajectories for larger protein systems consisting of several thousand atoms. One typical way to overcome these limitations is to use coarse graining. Here, the number of degrees of freedom is significantly reduced by combining multiple atoms into one “super-atom” or “bead”.

We used coarse grained (CG) MD simulations to study a chain of ubiquitin (Ub) proteins. Ub consists of 76 amino acids and plays an important role in cellular signaling. In a process called “ubiquitylation” an isopeptide bond is formed between a lysine group of a substrate protein and the C-terminal carboxylate group of an Ub molecule. Starting from this first Ub molecule other Ub moieties can be attached to form poly-ubiquitin chains (Ub-chains) of various lengths. The first attached ubiquitin offers eight potential linkage-sites: the N-terminal methionine (M1) and seven lysine residues (K6, K11, K27, K29, K33, K48, K63). Depending on the involved linkage-sites, chain length and topology, Ub-chains signal their substrate proteins for different functions, e.g., DNA damage tolerance or proteasomal degradation. (Pickart and Eddins, 2004; Komander and Rape, 2012).

**FIGURE 1:**
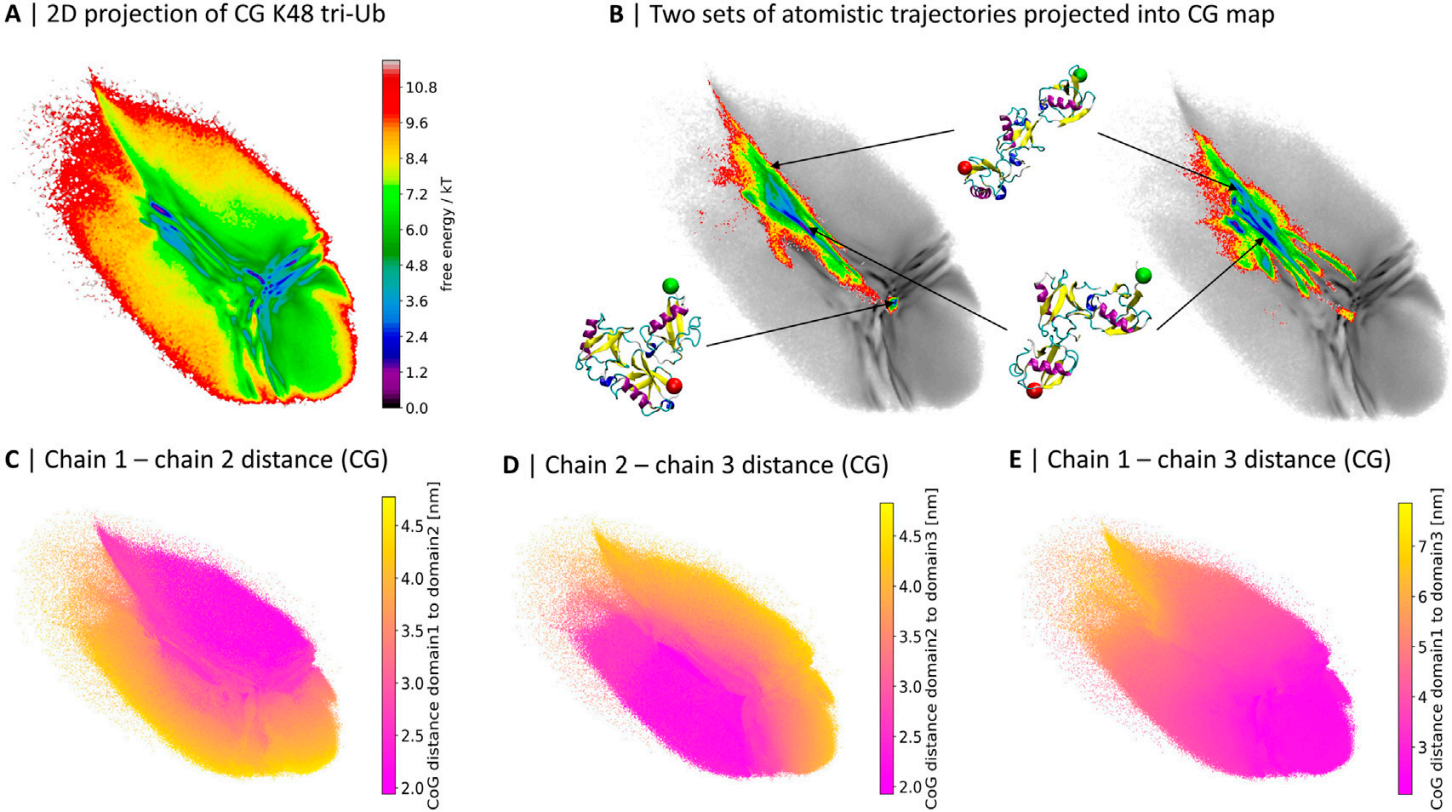
2D projections of K48-linked tri-Ub trajectories from coarse grained (Berg et al. (2020)) **(A)** and two independent sets of atomistic simulations **(B)**. **(B)** The atomistic simulations are colored based on free energy values, the CG map is gray and the same as in **(A)**; three exemplary conformations from the atomistic simulations and their location in the map are illustrated. A red sphere is attached to the first residue, indicating the proximal unit, and a green sphere is attached to the last residue, indicating the distal moiety. **(C–E)** 2D map colored by the center of geometry (CoG) distance between two of the three Ub moieties in the CG simulations.

To understand and explain differences in the physiological behavior of polyubiquitin chains one needs tools to characterize their conformational space. This is a challenging task due to a very dynamic behavior of Ub-conjugates and their conformational diversity. Thach et al. (2016) CG MD simulations in combination with dimensionality reduction and clustering techniques can be used to obtain a detailed description of the statistical ensemble of configurations populated by Ub-chains. Recently Berg et al. (2020) used a modified MARTINI v2.2 (Marrink et al., 2007; Monticelli et al., 2008; de Jong et al., 2013) CG force field and machine learning to describe and compare conformational spaces of di- and tri-Ub linked *via* all eight linkage-sites as well as free ubiquitins. Coarse graining massively speeds up the exploration of the phase space, but can potentially lead to inaccuracies. To assess the results of the CG sampling of tri-Ub we conducted extensive atomistic simulations (4 µs of simulation time in total) of K48-linked tri-Ub-chains starting from an extended conformation. We compared the phase spaces of CG and atomistic simulations by projecting all data to the same two-dimensional space (see Figures 1A,B, details on the projection method are given in Section 2).

Already at first sight, the comparison reveals that while the atomistic proteins quickly evolved from the extended starting conformation to more compact structures with contacts between the Ub-domains, large parts of the CG conformational space was not sampled during the 4 µs of atomistic simulations.

Out of the 40 brute-force atomistic simulations only two sampled the area in the middle of the map, corresponding to a completely collapsed conformation (see Figure 1B). In order to get a better understanding of the meaning of the different regions of the map, in particular those visited by the CG model but not the atomistic one, we colored the projection of the CG simulations based on the pairwise distance between the centers of geometry (CoG) of the three Ub moieties (Figures 1C–E). The conformational landscape can roughly be divided into three parts, which are separated by a “T”-like shape of more frequently sampled areas: the upper-right part represents conformations where the first and second Ub moieties are in close contact; the lower-left side contains conformations with close contacts between the second and third moieties; and lastly there is a gradient in terms of the distance between the first and the third moiety from the upper-left hand side to the lower-right hand side.

Now the question arises whether the fact that the atomistic simulations do not visit substantial parts of the CG conformational space results from insufficient length of the atomistic simulations or unphysical conformations produced by the CG model. One method that is very well suited to address this question is back-mapping based sampling (BMBS) (Hunkler et al., 2019). We introduced this technique by analysing a rather drastically coarsened model of oligopeptides. The application of BMBS allowed to reintroduce atomistic and dynamic information to the studied systems as well as to correct inaccuracies in the CG sampling. The core idea behind the method is the following: by navigating in two-dimensional free energy landscapes of very efficiently produced CG ensembles, selected conformations can be back-mapped to higher (e.g., atomistic) resolution to start new short explorative atomistic simulations in order to sample all of the accessible phase space as fast as possible. The convergence/divergence of the initial CG and obtained BMBS-guided atomistic landscapes is monitored quantitatively using a selected metric (earth mover’s distance (EMD) (Applegate et al., 2011)). Details are given in Section 2.2 and (Hunkler et al., 2019).

In the following we show how the BMBS algorithm can be used to resolve the question whether the discrepancies between the CG and atomistic landscapes stem from insufficient atomistic sampling or from a major flaw in the CG model. Moreover, we demonstrate here that BMBS is applicable to much larger systems compared to the ones it was introduced on. We extend the originally introduced BMBS scheme with analysis of the influence of the initial weights/biases of the back-mapped configurations used to start the atomistic BMBS simulations. We also perform detailed analysis of the atomistic ensemble obtained with BMBS applying a newly introduced clustering scheme Hunkler et al. (2022).

## 2 Methods/Computational details

### 2.1 Simulation details

All atomistic simulations were performed using either the 2016.4 or the 2020.4 version of the GROMACS package (Bekker et al., 1993) with a modified GROMOS 54A7 force field (Schmid et al., 2011) and the SPC/E water model. The force field was altered by the introduction of an isopeptide bond, to be able to simulate the covalently linked Ub moieties. Furthermore the following settings were used: the time step was set to 2 fs, the temperature was set to 300 K using the velocity rescale thermostat and the pressure was set to 1 bar with the Parrinello-Rahman barostat. As an integrator algorithm, the leap-frog algorithm was used. Long range interactions were computed with the particle mesh Ewald method, where a Fourier grid spacing of .16 nm and a pme-order of 4 were used. For Coulomb and van-der-Waals interactions, a cutoff of 1.4 nm was used. In order to constrain all bonds, the LINCS algorithm was applied.

For the CG simulations a modified MARTINI force field was used (based on MARTINI v2.2) (Marrink et al., 2007; de Jong et al., 2013) where protein-water interactions were increased to avoid proteins being too sticky. The MARTINI non-polarizable CG water was used as the solvent. The temperature was set to 300 K using the velocity rescale thermostat, pressure was kept at 1 bar by the Parrinello-Rahman barostat. The Verlet cut-off scheme was applied, the LINCS algorithm was utilised for bond constraining and the leap-frog integrator was used. A 10 fs time step was used due to the soft elastic network potentials (IDEN) (Globisch et al., 2013). The cutoff distance for short-range van-der-Waals interactions was set to 1.1 nm, and electrostatics were treated by the reaction field method with a cutoff distance of 1.1 nm and a dielectric constant of 15. For more details on how the MARTINI force field was modified see Berg et al. (2018).

### 2.2 Back-mapping based sampling

The back-mapping based sampling (BMBS) algorithm (Hunkler et al., 2019) was used to efficiently reintroduce atomistic resolution to CG simulations and is shortly summarised here. BMBS uses a low-dimensional projection of CG free energy surfaces to initiate new atomistic simulations and consists of the following steps: 1) CG simulations are projected to a two-dimensional landscape; 2) a number of selected CG structures are back-mapped to full resolution atomistic level; 3) new short atomistic simulations are run from the selected structures to rapidly explore the phase space; 4) convergence or divergence is monitored by comparing CG and atomistic probability distributions in low-dimensional space. Those steps rely on five main components: high-dimensional collective variables (CVs) applicable to both CG and atomistic configurations, a dimensionality reduction scheme, a method to select starting configurations from the CG ensemble (seeding), a back-mapping strategy and a statistical metric to monitor convergence. All of them are described below.

#### 2.2.1 Collective variables: Residue-wise minimal distances

In principle many different CVs/feature sets can be used in combination with the BMBS workflow. The specific choice of a CV is almost exclusively dependent on the given system. The only requirement regarding the CV is that it has to be able to describe the system in both resolutions (in the atomistic and the CG model). Therefore it must rely on coordinates that are present in both models. The CVs which we use here to describe and analyse the tri-Ub system are the residue-wise minimal distances (RMD). It has been shown before that the RMD are very well suited to describe the domain-domain configurations in ubiquitin chains since they are sensitive to the protein interfaces and to the distances and relative orientations of the domains (Berg et al., 2018; Berg and Peter, 2019; Berg et al., 2020). For one conformation of tri-Ub such a CV is a 432 dimensional vector, which contains the minimal distances of each of the 72 C_*α*_ atoms (the highly flexible residues 73–76 of ubiquitin were not considered) of each Ub domain to any C_*α*_ atom of each of the other moieties. This set of internal coordinates describes a distance as well as a relative orientation of individual ubiquitin moieties towards each other and can be applied to both atomistic as well as CG systems (if a backbone bead is present at any C_*α*_ location).

In order to describe the RMD vector of tri-Ub, the distal, middle and proximal moieties are abbreviated as A, B and C. In this notation “proximal” refers to the moiety with a free C-terminus with which the chain can be linked to the substrate and “distal” denotes the terminal moiety which is linked by its C-terminus to the middle Ub-unit. These three domains can be formulated as *A* = (*a*_1_, *a*_2_, *a*_3_, … , *a*_*n*_), *B* = (*b*_1_, *b*_2_, *b*_3_, … , *b*_*m*_) and *C* = (*c*_1_, *c*_2_, *c*_3_, … , *c*_*o*_), where *a*_*i*_, *b*_*j*_ and *c*_*k*_ are positions of the C_*α*_ or the backbone beads respectively. Then pairwise distance matrices *D*_*A*,*B*_, *D*_*B*,*C*_ and *D*_*A*,*C*_ are computed. By taking the minimum values in each respective row and column the vectors of the residue-wise minimum distances between all three moieties (*A*_*B*_, *B*_*A*_, *B*_*C*_, *C*_*B*_, *A*_*C*_, *C*_*A*_) are calculated. Those vectors are then concatenated to one high-dimensional representation (432 dimensions) of the considered tri-Ub conformation, the RMD vector. All CG configurations are projected to two dimensions by using their RMD vectors as input features for the dimensionality reduction method encodermap (Lemke et al., 2019; Lemke and Peter, 2019).

#### 2.2.2 Dimensionality reduction: Encodermap

Encodermap (Lemke et al., 2019; Lemke and Peter, 2019) utilizes an autoencoder architecture but adjusts the autoencoder loss function by adding a multidimensional-scaling-like loss term [Equations 1 to (Eq. 3)]. This additional loss function transforms all distances by a sigmoid function (Eq. 4) and is termed as sketch-map loss due to its connection to the sketch-map dimensionality reduction method Ceriotti et al. (2011). The sketch-map loss function enables encodermap to reproduce the connectivity between high-dimensional data points in a 2D map, meaning that conformations with similar high-dimensional CVs are also located close to each other in the 2D projection. Furthermore, the autoencoder architecture enables the method to project huge amounts of data in a very short time.

$$L_{\mathrm{encodermap}}=k_{a}L_{\mathrm{auto}}+k_{s}L_{\mathrm{sketch}}+Reg$$ (1)

$$L_{\mathrm{auto}}=\frac{1}{N}\overset{N}{\underset{i=1}{\sum }}D\left(X_{i},{\tilde{X}}_{i}\right)$$ (2)

$$L_{\mathrm{sketch}}=\frac{1}{N}\overset{N}{\underset{i\neq j}{\sum }}{\left[SIG_{h}\left(D\left(X_{i},X_{j}\right)\right)-SIG_{l}\left(D\left(x_{i},x_{j}\right)\right)\right]}^{2}$$ (3)

Here, *k*_*a*_, *k*_*s*_ are adjustable weights, *Reg* is a regularization used to prevent over-fitting; *N* denotes the number of data points to be projected; *D* (⋅, ⋅) is a distance between points, *X* is the high-dimensional input vector, *x* is the low-dimensional projection (the bottleneck layer); *SIG*_*h*_ and *SIG*_*l*_ are sigmoid functions of the form shown in Eq. 4,

$${SIG}_{\sigma ,a,b}\left(D\right)=1-{\left(1+\left(2^{\frac{a}{b}}-1\right){\left(\frac{D}{\sigma }\right)}^{a}\right)}^{-\frac{b}{a}},$$ (4)

where *a*, *b* and *σ* are parameters defining the range of distances to preserve.

Once the network has been trained, the encoder works as a mathematical function that maps the high-dimensional inputs to the low-dimensional projection. In this mapping function lies one of the main advantages of the encodermap algorithm, namely the extremely efficient projection of additional high-dimensional input data points to the low-dimensional space.

Since the encodermap method is non-linear, the axes of the resulting 2D space do not necessarily allow a physical interpretation in terms of order parameters. Therefore we chose to omit the x- and *y*-axes for all 2D plots shown in this manuscript. Adding these axes would in our opinion rather mislead the reader than help in understanding the figures.

Similar to the choice of CVs, a different dimensionality reduction method can be chosen to be used with the BMBS workflow. However, such a method should fulfill a few requirements. First it has to be possible (and preferably fast) to project additional data points to the low-dimensional space. And secondly the method should be able to separate different structures reliably in the low-dimensional space (2D or 3D if one wants to visualize the projection). Encodermap performs remarkably well in both of these tasks and is extremely efficient in projecting data once it is trained.

The parameters for encodermap used in this work are given in Table 1. We used encodermap version 2.0.1 and its implementation from https://github.com/AG-Peter/encodermap.

**TABLE 1:** Encodermap parameters used to generate the 2D projection shown in this work.

Encodermap parameters	*N*_*steps*_	*N*_*layers*_	*N*_*neurons*_	*σ*_*highD*_	A	B	*σ*_*lowD*_	a	b	*k*_*a*_	*k*_*s*_
Values	10,000	3	300	20	12	10	1	2	10	1	500

#### 2.2.3 Seeding

**FIGURE 2:**
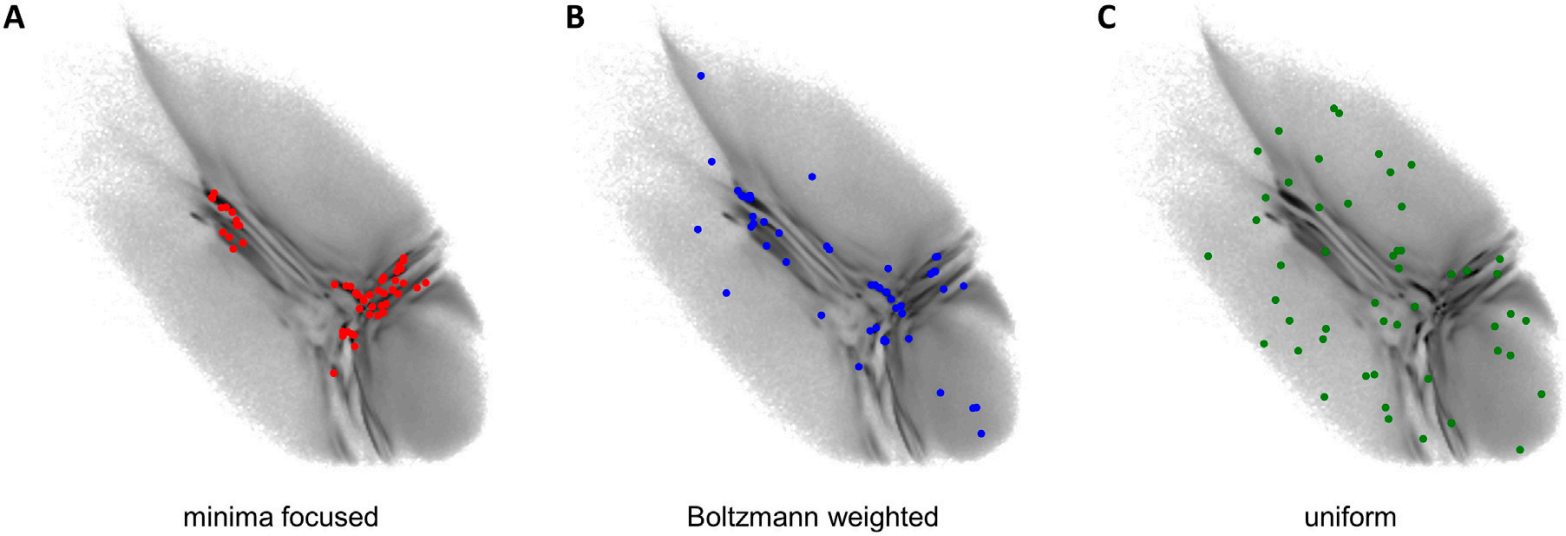
Seeding strategies used in this paper: **(A)** minima focused (red points), **(B)** Boltzmann weighted (blue points), **(C)** uniform (green points). Each seeding consists of 50 back-mapped conformations. Their projections are shown on the original CG landscape (gray gradient, same as in Figure 1 **(A)**).

The obtained two-dimensional projection of the CG ensemble is used to seed new short atomistic MD simulations from back-mapped CG structures. If the starting conformations are chosen properly, it takes the BMBS simulations only a fraction of the simulation time compared to a standard MD to sample a comparable amount of the available phase space. In the original BMBS paper Hunkler et al. (2019) the starting configurations were chosen based on the minima in the two-dimensional CG landscape (Figure 2A). In this paper we want to explore in more details different seeding strategies and study their influence on the BMBS performance. In addition to the original seeding method, which we call here minima-focused, we test Boltzmann-weighted and uniform seeding (see Figure 2).

For the minima-focused seeding we chose the starting structures to replicate the deepest free-energy minima of the CG 2D distribution and their weighting as well as possible. To achieve this we applied a binning to the 2D CG space and created a list with the most populated bins. Then we randomly chose a data point from the highest populated bin and repeated this until the percentage of starting structures from this bin approximately matched the percentage of data points in this bin. This procedure was reiterated for all the most populated bins until a predefined number of starting conformations (50 in this paper) were obtained.

The Boltzmann-weighted seeding was chosen to also include rare conformations in the starting structures. We binned the 2D space as before but randomly picked one bin and accepted or rejected this bin with a Monte Carlo criterion (a probability proportional to the bin’s population). A random data point from the accepted bin was chosen as a starting structure and the process was repeated until 50 data points were selected. Such a procedure allowed us to include rare conformations and retain as well as possible the original CG distribution given a very limited sample size (50 points). Theoretically with a much larger sample size this procedure would converge to a random selection of starting configurations from the full high-dimensional configuration space.

Lastly we chose a uniform seeding (with uniform referring to a uniform distribution in the 2D space). We again used the same binning as before and randomly chose one bin. From this bin one data point was randomly selected and the bin was then removed from the pool of available bins (the removal of a bin becomes important if the number of chosen data points approximates the number of available bins). This was again repeated until 50 starting points were selected.

The results of different seedings are compared in Section 3.1.

#### 2.2.4 Back-mapping

In the main part of the original paper the back-mapping was done by taking an atomistic structure with CVs similar to a target CG structure. Then an external restrictive potential was applied to the atomistic structure during an energy minimization step in order to force its conformation to retain the CVs of the CG target. In this work we used CG trajectories generated with the MARTINI model and thus applied the “backward” (Wassenaar et al., 2014) script to reintroduce an atomistic resolution into selected CG structures.

#### 2.2.5 Statistical metric: Earth mover’s distance

To monitor a similarity between two conformational phase spaces, e.g., a CG and atomistic sampling, we use the earth mover’s distance (EMD) (also known as Wasserstein’s metric or Mallows distance). It is a metric that describes how similar or dissimilar two given multivariate distributions are. For a formal definition of the method see e.g., Applegate et al. (2011). In order to be able to quantitatively compare the EMD values we use unity-based normalized EMDs. This implementation of the EMD brings all values into the range (0,1) (Eq. 5).

$$EMD^{\prime }=\frac{EMD-\min\left(EMD\right)}{\max\left(EMD\right)-\min\left(EMD\right)},$$ (5)

with min(*EMD*) = 0 and max(*EMD*) = 1.62. The coefficient max (*EMD*) is hereby defined as the EMD for the comparison of the CG 2D projection with a uniform rectangular 2D distribution with the same amount of data points. The dimensions of this 2D rectangular area are given by the minimum and maximum *x* and *y* values of the CG projection. By implementing the EMD in such a way, a value of 0 means that two given distributions are exactly identical and a value of 1 means that two distributions are as dissimilar as the CG projection compared to a uniformly distributed data set. In order to compute the EMDs we used the python implementation *pyemd* v0.5.1 (Pele and Werman, 2009).

### 2.3 Clustering scheme

To analyse atomistic ensembles of such complex systems as tri-Ub we use a recently introduced clustering scheme which can effectively work with large amounts of high-dimensional data Hunkler et al. (2022). In this iterative clustering workflow we use HDBSCAN (Campello et al., 2015) as the clustering algorithm and combine it with two different dimensionality reduction algorithms, namely cc_analysis (Diederichs, 2017) and encodermap (Section 2.2.2). HDBSCAN is a hierarchical density-based clustering algorithm which is able to find clusters of different shapes and densities requiring only a small number of input parameters (at least one). The cc_analysis is a multidimensional-scaling-like method that minimizes the differences between Pearson correlation coefficients of high-dimensional data points and the scalar product of low-dimensional vectors representing them.

In this clustering workflow the probability density in the cc_analysis projection is used as the clustering space (intermediate dimensionality; usually between 10 and 40 dimensions), while the 2D encodermap space is utilized to efficiently process large data sets and assign additional conformations to already identified clusters. The provided data set is clustered iteratively until a specified amount of conformations is assigned to clusters or until a specified amount of clustering iterations have been performed. In the process of assigning conformations to clusters a root-mean-square deviation (RMSD) cutoff of C_*α*_ atom positions is used to obtain conformationally very defined clusters.

For applying the clustering scheme to the tri-Ub system we set the HDBSCAN parameters “min_cluster_size” and “min_samples” to 80 and used an RMSD cutoff distance of 3 Å. The clustering scheme was run for three iterations.

## 3 Results and discussion

### 3.1 BMBS

**FIGURE 3:**
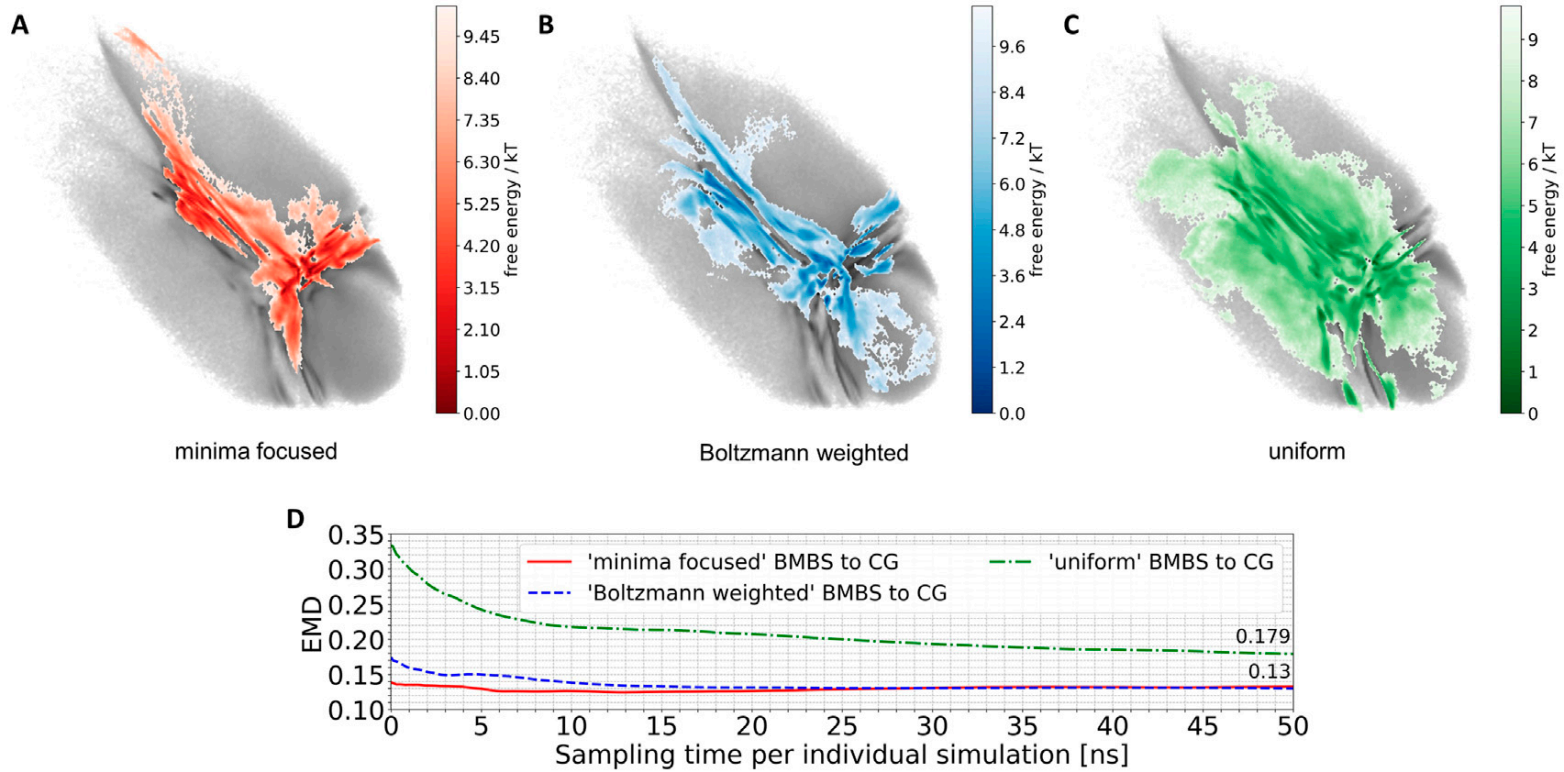
Projections of 50 atomistic simulations obtained using BMBS with minima-focused **(A)**, Boltzmann-weighted **(B)** and uniform **(C)** seedings. **(D)** EMD values between CG and BMBS projections as a function of sampling time. Colors of the projections and EMDs lines correspond to the coloring in Figure 2.

We applied the BMBS method to the K48-linked trimer of ubiquitin with three different seeding algorithms: minima focused, Boltzmann weighted, and uniform (see Section 2.2.3 for detailed description). In each case we chose 50 starting points. For every starting structure we ran an atomistic MD simulation for 50 ns with a cumulative simulation time of 2.5 µs for each seeding. The location of the 50 starting points is shown in Figure 2. The BMBS simulation trajectories were projected to the original CG landscape and can be seen in Figures 3A–C. These three maps show that the choice of starting points heavily influences the resulting conformational space (a detailed analysis of the obtained conformations and their spreading in the 2D projections is discussed in Section 3.3).

The BMBS with all three seedings visited the bottom part of the CG 2D map which was not sampled by the two initial 4 µs atomistic simulations (compare to Figure 1B). Notably the uniformly seeded trajectories retain the “T” shaped arrangement of free-energy minima of the original distribution even though only few of the starting conformations were selected in those parts of the map. This indicates a rather quick progression of the trajectories that were seeded near the rims to the center part of the 2D projection.

A purely visual comparison of the obtained maps can be misleading as it is important to not only cover the CG phase space but to properly sample the free energy minima. For a quantitative comparison of such two-dimensional distributions we use the EMD, which fits perfectly into the BMBS workflow. The EMD is not sensitive to bin sizes (can be applied for comparing different histograms), is symmetric, and is more sensitive to similarities in highly populated regions than to the rarely populated ones. The EMD values comparing the original CG projection with the time evolution of the differently seeded BMBS projections are shown in Figure 3D. Contrary to visual perception, the EMD plot shows that both the minima-focused and Boltzmann-weighted seedings produce atomistic ensembles whose projections resemble the CG target map much better (an EMD value of .13 after 50 ns of simulation time of the individual runs) than the projection of the uniformly seeded trajectories (.179). On the other hand, the uniformly seeded BMBS approaches the CG distribution very quickly, especially in the first 10 ns of individual simulation time. To put these EMD values into perspective, the comparison of the projection of the initial 4 µs atomistic simulations to the CG distribution gives an EMD of .815.

Therefore we can address the initial question on the reason of the discrepancy between the CG and atomistic ensembles. By applying the BMBS algorithm to the K48-linked tri-ubiquitin, we obtained 150 atomistic BMBS trajectories which provide enough evidence to confidently say that the CG ensemble does not include a large amount of unphysical conformations. Given enough simulation time, the two initial atomistic trajectories would most likely also have sampled the conformations that reside in the lower parts of the 2D map.

The generation of these new atomistic trajectories is however only one aspect of the BMBS algorithm. Another part is the monitoring and comparison of the 2D histograms which develop over time. This analysis is provided in the next section.

### 3.2 EMD monitoring

**FIGURE 4:**
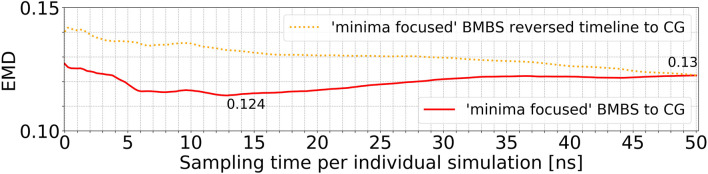
Time resolved EMDs of both forward (red solid line, same as in Figure 3D) and reversed (orange dotted line) timelines of minima-focused BMBS histograms to the CG map.

In order to analyse the temporal/chronological development of the BMBS compared to the CG map we extracted 2D projections of BMBS trajectories for different sampling times. We chose to generate one histogram every 250 ps of individual simulation time for a good temporal resolution. This resulted in 200 projections for each seeding approach. For each of these histograms we computed the EMD to the CG 2D map and obtained EMD values shown in Figure 3D and Figure 4.

In addition to the time evolution of the minima-focused BMBS (red lines in both figures) provided in Figure 3D, Figure 4 shows the reversed timeline of the minima-focused BMBS histogram (orange line) to the CG map. By reversed we mean that the projection of the last frame of each minima-focused trajectory is the starting point from which the histogram grows contrary to the original timeline, meaning that each histogram starts from a point where the trajectory could sample for some time and therefore will most likely be in some meta stable state. The forward timeline (red line in Figure 4) has a non-monotonic behaviour with the initial decrease in EMD values (the two histograms become more similar to each other) until about 13 ns, followed by an increase and plateauing of the values at about .13. The same behaviour was found in the original (Hunkler et al., 2019) paper for a predictive CG model based on extrapolated data and could be explained as a correction of flaws in CG sampling. To reduce the influence of the seeding bias on the 200 time-resolved histograms we also included the reversed timeline (orange line in Figure 4). This timeline shows that the BMBS trajectories moved away from their initial seedings. With increasing simulation time the trajectories approach their original starting points, which leads to a decrease in the EMD values. This clearly shows that the BMBS trajectories move away from the most populated areas in the CG 2D map and indicates that the underlying CG distribution of conformations is not perfectly representing the conformational ensemble corresponding to the atomistic Hamiltonian.

**FIGURE 5:**
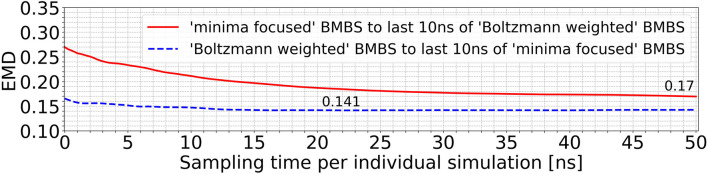
EMDs of the entire trajectories of the minima-focused seeding to the histogram of the last 10 ns of the Boltzmann-weighted seeding (red curve) and vice versa (blue curve).

Using EMDs we also monitored and compared the behaviour of different seeding approaches to each other. Figure 5 compares the minima-focused (red curve) and Boltzmann-weighted (blue curve) seedings to the histograms generated by the last 10 ns of the simulations from the respective other seeding. With this comparison we can identify if two sets of trajectories converge to sample a shared part of phase space or whether they diverge over time to different accessible areas of the conformational space. The blue curve in Figure 5 changes only slightly, while there is a much more significant decrease in the red curve. The minima-focused histograms are more similar to the histogram representing the last 10 ns of the Boltzmann-weighted trajectories than vice versa (reflected by the generally lower EMD values). These observations allow us to draw two conclusions. First, the minima-focused trajectories initially move away from their seeding points but then do not change much in the remaining simulation time. And secondly, the Boltzmann-weighted trajectories significantly move away from their original seeding and approach the same areas in the 2D map as the minima-focused trajectories. This shows that the two systems evolve in the same general direction, even though they are partially sampling quite different areas of the 2D map at the end of the simulations.

Lastly we assess the question if the convergence of MD simulations can be monitored using EMDs. Generally, a continuous upwards or downwards trend in the EMD values indicates that the corresponding atomistic ensemble has not converged yet. However, even if the EMD curve has not changed significantly over a longer period of time, that does not imply that a convergence has been reached. As can be seen in Figure 3D the EMD plots from 25 to 50 ns of individual simulation time for all three seedings only show a very minimal change over time. But by comparing the three curves quantitatively, one observes higher EMD values for the uniform seeding compared to other two approaches, consequently the uniform simulations cannot be converged. Overall this means that none of the three BMBS ensembles can be considered converged and that an additional simulation time has to be invested to cover the full phase space and produce an ensemble that is representative of the actual atomistic free-energy landscape. However, the EMD of 2D histograms can be an additional easily employed and efficient indicator of the current degree of non-convergence.

The general workflow which we propose in this manuscript is compatible with any atomistic force field, water model or CG model (as long as the CV of choice is available in both the atomistic and CG representations). In Hunkler et al. (2019) we demonstrated the use of the BMBS with different CG models, moreover it can be very informative in comparing the 2D probability distributions of various atomistic or coarse grained force fields with each other. As an example one could take the results of the comparison of the probability distributions generated by the two force fields used in this work (modified GROMOS 54A7 and modified Martini v2.2). We have shown that the resulting 2D distributions differ and have interpreted this difference as flaws in the CG model (i.e. due to the shape of the minima-focused EMD curve). Yet, it would be difficult to prove whether the discrepancies in the 2D projections actually stem from the CG or the atomistic model (or both). If however, we would now make the same comparison using a different atomistic force field (but the same back-mapped starting conformations), we could compare both the atomistic 2D distributions with the CG model, as well as the atomistic distributions with each other. This could lead to a much better understanding of the origin of the differences in the 2D projections and be useful for efforts to improve simulation models in either resolution.

To summarize, the EMD, especially if used in a time resolved fashion, is a very useful tool to analyse (2D) projections of the sampled phase space of MD trajectories. We showed that the EMD can be used to follow atomistic trajectories (that were specifically seeded based on the minima of a CG template map) evolution over time compared to the CG template. By first approaching the seeding template but then moving away from it, the EMD curve alludes to a correction of flaws in the CG map. This assessment of the quality of the CG model is one of the strongest features of a minima-focused back-mapping based sampling. The uniform seeding on the other hand is primarily useful in order to obtain atomistic conformations from all the CG space as fast as possible. However, if one wants to generate a (close to) converged atomistic ensemble that realistically represents the actual conformational landscape, the Boltzmann-weighted seeding is the best choice. It is on the one hand much faster in sampling of low energy conformations compared to the uniform seeding (assuming the CG model is somewhat viable) and on the other hand it includes less bias of the CG map compared to the minima-focused seeding.

### 3.3 Cluster analysis

For the choice of starting configurations and the monitoring of the convergence, the BMBS scheme relies on the 2D projection of the CG configurational space. This is a radical reduction in dimensionality considering the size of tri-Ub. Thus we decided to assess a quality of this map by performing a clustering analysis in the high-dimensional space of the atomistic configurations sampled with BMBS. Such clustering can provide information on general conformational trends in the map (similar to the change in CoG distances between Ub moieties shown in Figures 1C–E) or show if the 2D projection is able to separate relatively similar conformations. Additionally it allows us to study the behaviour of individual short trajectories, e.g., whether the same conformations were sampled by trajectories from different origins (i.e. different seeding schemes and different starting regions on the 2D map). This can complement the convergence analysis based on the EMDs discussed in Section 3.2. Considering the system sizes and complexity we used a recently developed clustering scheme which is specifically designed to efficiently cluster large MD trajectories Hunkler et al. (2022) (see Section 2.3).

**FIGURE 6:**
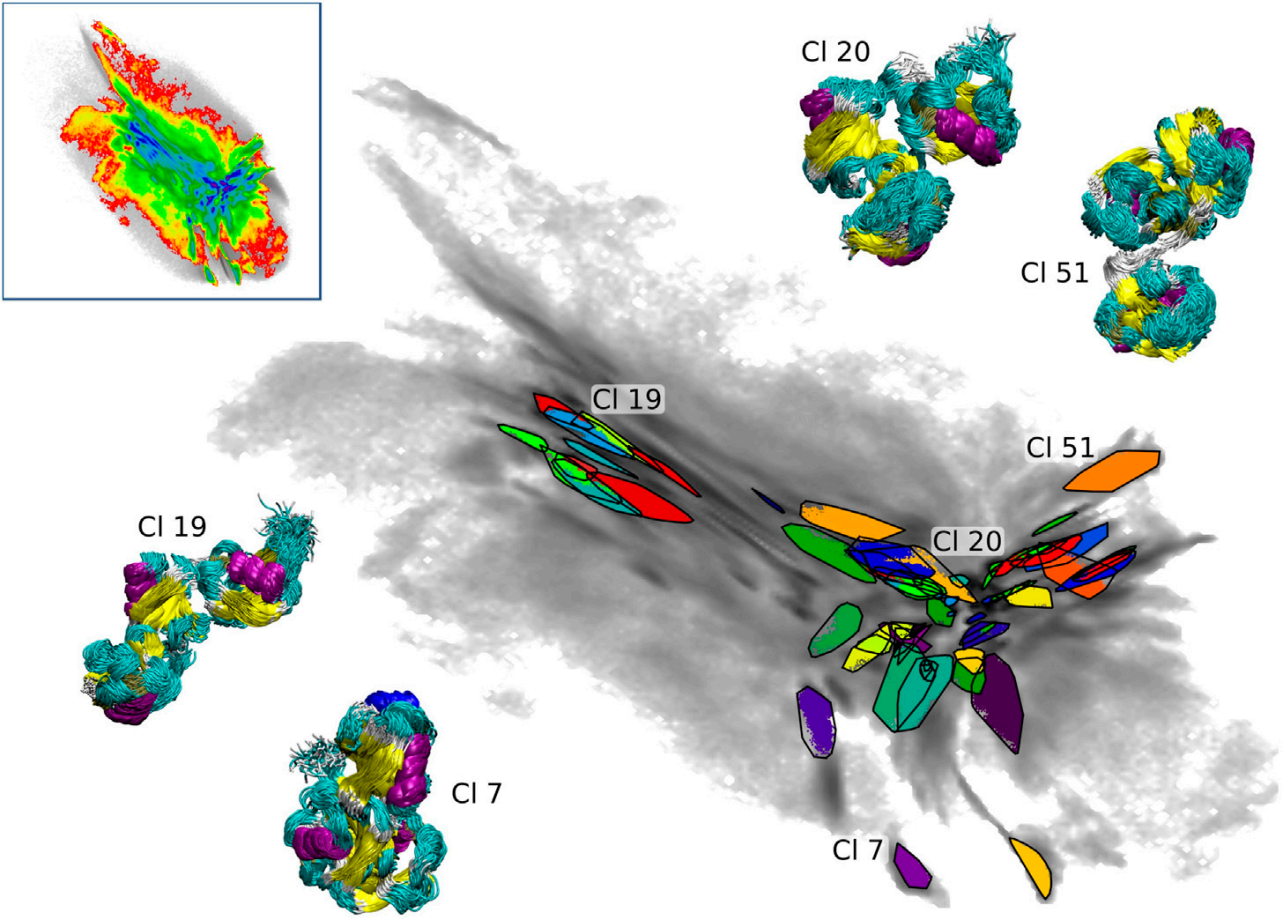
Projections of 61 clusters from the combined BMBS trajectories (gray gradient). Bundles of the structures (colored according to the secondary structure) from selected clusters are shown to visualize the homogeneity of the found clusters. The upper left inset shows the projection of the combined BMBS trajectories in the original CG landscape.

We applied the clustering workflow to the combined atomistic data of all three seeding schemes (upper left inset in Figure 6). The data set contains 7.44 million conformations and 30% of these were assigned to 61 clusters after three iterations of the clustering process (the RMSD cutoff was set to 3 Å). As described in details in Section 2.3, the clustering was performed in the intermediate-dimensional space determined by cc_analysis and the resulting clusters were then projected into the 2D map. They are shown in Figure 6 including tri-Ub structures belonging to four example clusters (structure bundles in the insets) to demonstrate the structural consistency obtained by the clustering method (the shown cluster numbers are used as they are assigned during the clustering process and do not reflect any meaningful ordering e.g., by cluster size). The compact placement of the clusters on the map shows that the 2D map is a meaningful representation of the high-dimensional conformational landscape - a property that was important for the use of this projection for BMBS and for the comparison of the atomistic and CG sampling with EMD.

**FIGURE 7:**
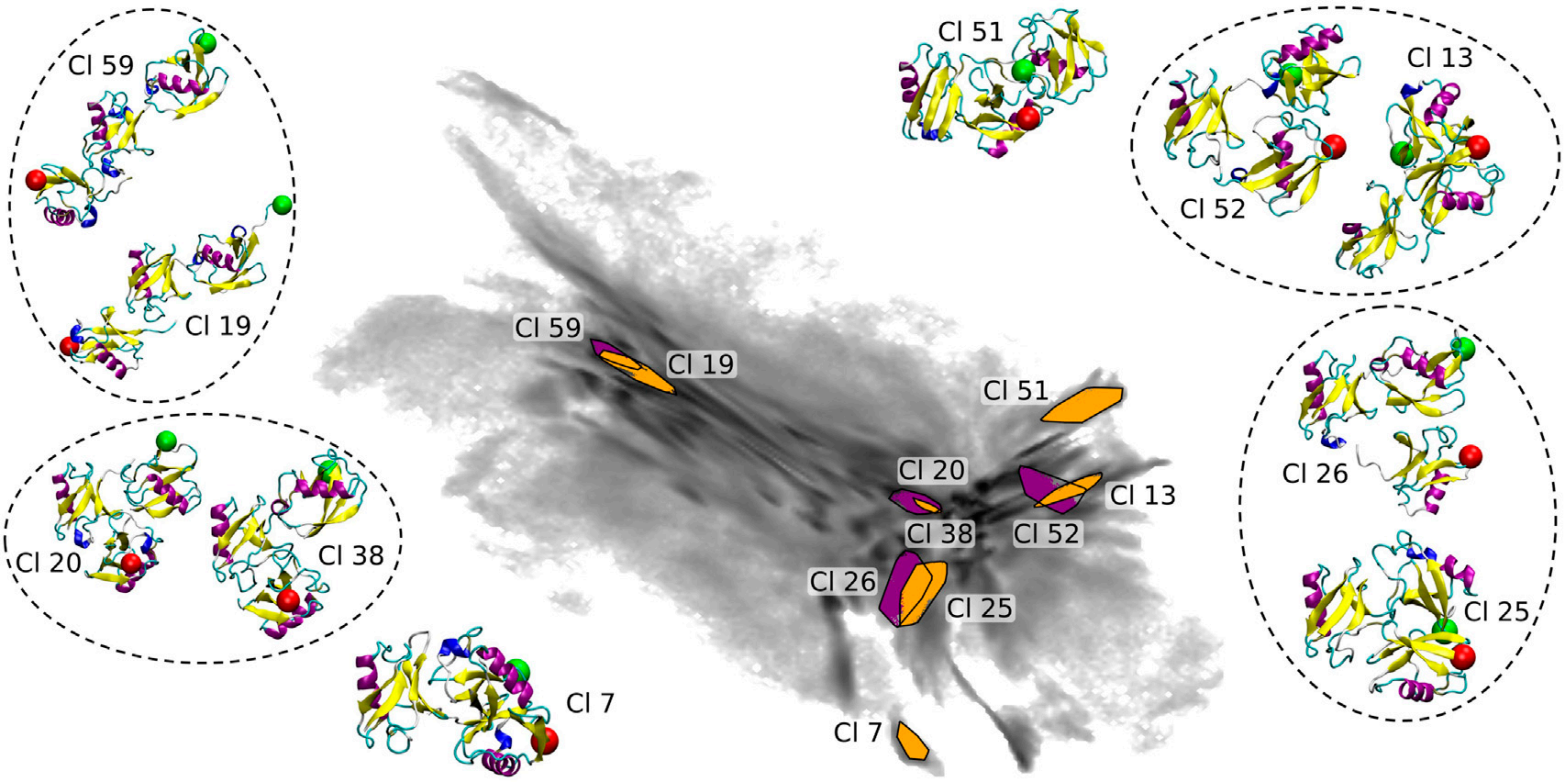
Selected clusters and their representative conformations in the BMBS projection (the same as in Figure 6). In all inset plots the middle moiety of the tri-Ub system is positioned in the same way. A red sphere is attached to the first residue, indicating the proximal unit, and a green sphere attached to the last residue, indicating the distal moiety. Conformations from clusters overlapping in 2D map are circled.

The coloring based on the CoG distances shown in Figures 1C–E provides a general understanding of the map. In order to get a more detailed insight we show 10 clusters (including representative tri-Ub configurations) from all parts of the 2D map (see Figure 7). These clusters were selected based on their location in the 2D projection. Conformations at the left hand side of the map (example clusters 19 and 59) are in general open chain conformations, meaning that the proximal and distal moieties extend to opposite directions from the middle moiety. The two clusters 20 (the largest cluster containing 3.5% of all conformations) and 38 in the center of the map adopt a collapsed conformation where each of the three moieties are roughly in equal distance to each other. Those are the most stable conformation in the system. One possible reason for this stability is that the hydrophobic patches on the distal and the middle moieties (primarily the part around the residues Ile 44 and Val 70) are orientated towards the other units and are thereby shielded from solvent. Cluster 38 intersects in the 2D projection with cluster 20. They are however still identified as two different clusters since they differ (mostly) in a small rotation of the distal moiety. This is a nice illustration of the precision and sensitivity of the proposed clustering workflow and its ability to pick up such minimal structural differences and separate the conformations into different clusters. Other examples of clusters overlapping in the 2D projection but having small structural differences identified by clustering in a higher-dimensional space are circled in Figure 7. In the clusters 51 and 52 (on the right hand side of the map) the middle and distal moieties (green sphere) are further apart than in the most populated cluster 20 (middle of the map). Especially in cluster 51, the proximal moiety is almost located between the other two. For cluster 7 the situation is exactly reversed, here the distal and middle chains are more distant and the proximal chain is located in between the two other units. So the clusters shown here confirm the general trends that we derived from the CoG distance distributions.

**FIGURE 8:**
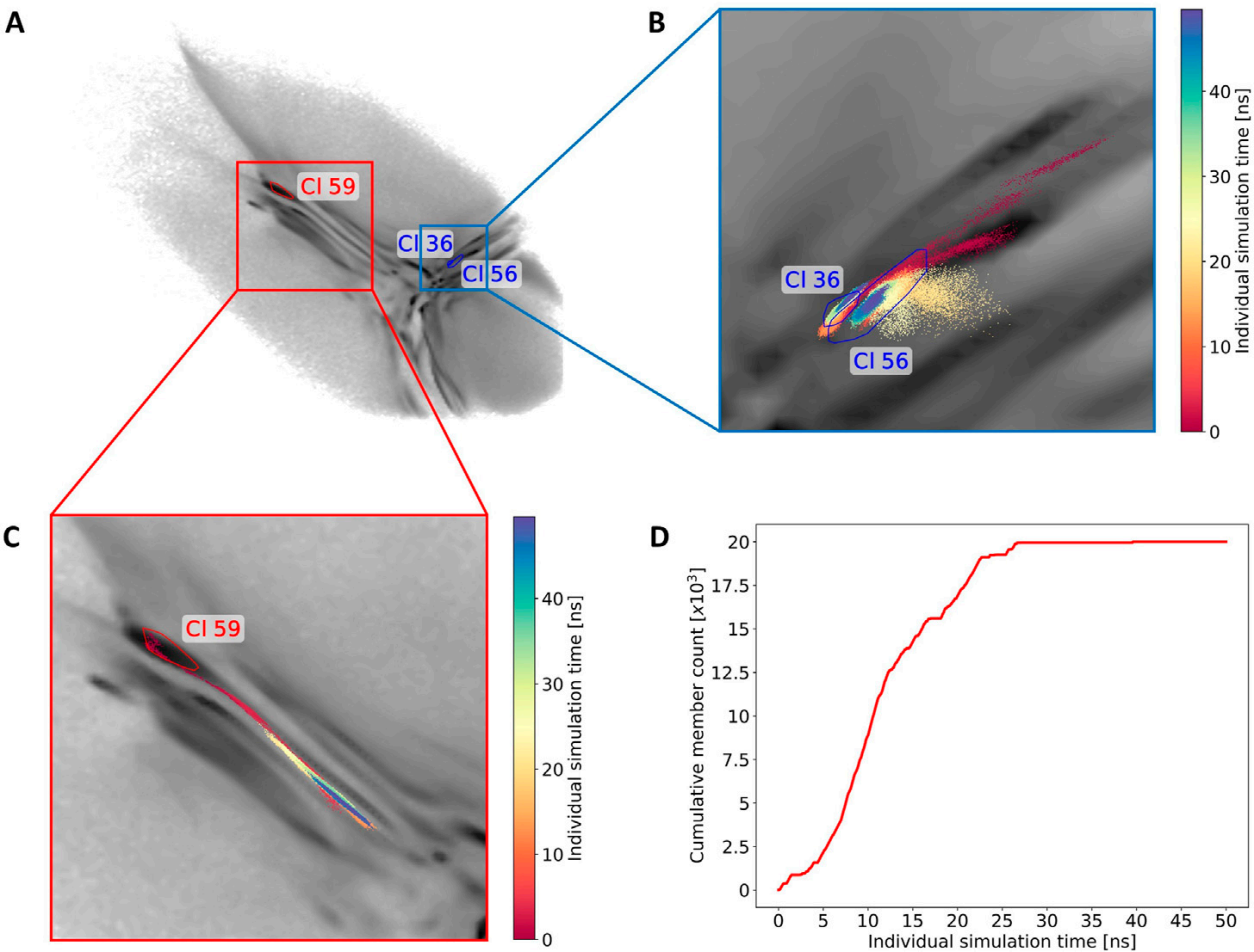
Monitoring of the sampling for three selected clusters. **(A)** The convex hulls of clusters 36, 56 and 59 in the 2D map. **(B)** A projection of one exemplary trajectory that contributes to the forming of clusters 36 and 56. The trajectory is colored based on the simulation time (staring from red to blue). **(C)** Example of one trajectory that leaves cluster 59. **(D)** The cumulative count of frames contributing to cluster 59 over the individual simulation time.

By using this clustering analysis we can also try to verify our statement about the ability of BMBS to correct flaws in the CG sampling using the minima-focused seeding. In Section 3.2 we argued (based on the minima-focused BMBS vs CG EMD plots) that the atomistic BMBS trajectories partially move away from the area in the 2D projection they were seeded in and thereby generate an atomistic 2D distribution that slightly differs from the CG one. This process can be seen as a mending of inherent defects in the CG model. To verify this, we inspect a few clusters and follow individual trajectories in the 2D landscape (Figure 8A). We start again with cluster 59 (left side of the map with extended conformations). Of the 150 independent trajectories 8 were initiated in or around that state but leave the cluster during the simulation time (a projection of one such trajectory is illustrated in Figure 8C). Figure 8D shows the cumulative number of members of cluster 59 *versus* the simulation time of the individual trajectories. This plot illustrates that the simulated trajectories indeed first sample cluster 59 and quickly populate it until around 11 ns of individual simulation time, but then the amount of conformations that are assigned to the cluster decreases. From around 25 ns onwards the cluster is not expanding. This means that after the first half of the simulated time all trajectories that have been initiated in this cluster (due to the high population of that specific area in the CG projection) have moved away from it. This example complements the correction trend observed in the EMD plots (Figure 4).

Next we show an example of two intersecting clusters 36 and 56 which are formed by several atomistic trajectories (Figure 8A). Figure 8B shows projections of two selected trajectories forming these clusters. In this case four BMBS trajectories that were initiated in and around a local minimum of the CG projection moved away from their seeding points and formed clusters in a less populated area of the CG map. This is another illustration where the 2D distribution of the atomistic BMBS trajectories slightly differs from the CG template distribution. This time, however, the BMBS trajectories do not collectively abandon one area of the map, but rather collectively move towards one specific section that was not heavily populated by the CG model.

## 4 Conclusion

We have applied back-mapping based sampling to obtain a conformational free-energy landscape of a flexible multidomain protein—K48-linked tri-ubiquitin—at atomistic resolution. BMBS had been introduced for much smaller peptides, where we had shown that the method is able to very efficiently generate a correctly weighted atomistic ensemble based on a 2D projection of a coarse grained simulation ensemble. For tri-Ub we first generated a 2D projection of a set of extensive CG simulations with the help of the dimensionality reduction method encodermap. From projecting the structures from a long (4 µs) atomistic simulation onto this 2D map, we found that these simulations had only visited a very limited part of the CG 2D landscape. By employing the BMBS algorithm, we found that the entire CG map is accessible to the atomistic trajectories, i.e. the CG simulations had in fact not sampled unphysical conformations. Rather, free energy barriers between different (metastable) conformational states are too high to be easily overcome on the timescales accessible to the atomistic model. This successful application of BMBS to tri-Ub illustrates that the method scales very well with system size. Furthermore we compared different seeding methods to initiate the atomistic simulations in the 2D projection: minima focused, Boltzmann weighted and uniform. We argue that Boltzmann weighted seeding is more advantageous in its ability to retain a correct free energy profile on the one hand and, on the other hand, to explore bigger areas of conformational space. In this context we also illustrate and discuss the use of the EMD metric for the comparison of different (2D) distributions in a time-resolved fashion. Lastly, we employed a recently introduced conformational clustering workflow to the combined atomistic BMBS trajectories. In doing so we illustrate which parts of the 2D map represent which structural conformations. In this context we also show that the encodermap algorithm separates different conformational characteristics very well into different regions of the 2D map, which validates the whole BMBS approach. Finally, we show how individual atomistic BMBS trajectories sample conformational states, move through the 2D map and in sum converge to an atomistic 2D distribution that slightly differs from the CG one, indicating a correction of flaws in the CG template.

## Data Availability

The python notebooks used to analyze the data in this study, as well as a minimal example consisting of 28,000 random CG structures can be found in https://github.com/AG-Peter/BMBS_of_tri-ubiquitin. The CG trajectories, selected back-mapping points and encodermap projections of all used data can be found in https://doi.org/10.48606/40.
